# Oxidation of KCNB1 channels in the human brain and in mouse model of Alzheimer’s disease

**DOI:** 10.1038/s41419-018-0886-1

**Published:** 2018-07-26

**Authors:** Yu Wei, Mi Ryung Shin, Federico Sesti

**Affiliations:** Department of Neuroscience and Cell Biology, Rutgers University Robert Wood Johnson Medical School, 683 Hoes Lane West, Piscataway, NJ 08854 USA

## Abstract

Oxidative modification of the voltage-gated K^+^ channel subfamily B member 1 (KCNB1, Kv2.1) is emerging as a mechanism of neuronal vulnerability potentially capable of affecting multiple conditions associated with oxidative stress, from normal aging to neurodegenerative disease. In this study we report that oxidation of KCNB1 channels is exacerbated in the post mortem brains of Alzheimer’s disease (AD) donors compared to age-matched controls. In addition, phosphorylation of Focal Adhesion kinases (FAK) and Src tyrosine kinases, two key signaling steps that follow KCNB1 oxidation, is also strengthened in AD vs. control brains. Quadruple transgenic mice expressing a non-oxidizable form of KCNB1 in the 3xTg-AD background (APP_SWE_, PS1_M146V_, and tau_P301L_), exhibit improved working memory along with reduced brain inflammation, protein carbonylation and intraneuronal β-amyloid (Aβ) compared to 3xTg-AD mice or mice expressing the wild type (WT) KCNB1 channel. We conclude that oxidation of KCNB1 channels is a mechanism of neuronal vulnerability that is pervasive in the vertebrate brain.

## Introduction

The imbalance between the production of reactive oxygen species (ROS) and the ability of the cells to detoxify them, referred to as oxidative stress, is a hallmark of aging and a number of pathologies^1^. One protein known to undergo oxidation in the brain is the voltage-gated K^+^ channel subfamily B member 1 (KCNB1)^2^. ROS induce cross-linking of KCNB1 subunits to each other (oligomerization) via the formation of disulfide bridges. Once oxidized, KCNB1 channels do not conduct current and accumulate in the plasma membrane from where they activate an outside-in signaling pathway mediated by integrins—with whom they form macromolecular complexes—focal adhesion kinases (FAK), Src tyrosine kinases and c-Jun N-terminal (JNK) kinases^3,4^. The concerted action of these kinases stimulates the production of more ROS and induces apoptosis. Traumatic brain injury (TBI) has provided a good model to assess the consequences of KCNB1 oxidation in vivo, because oxidative stress is extensive in this condition^5,6^. Transgenic mice overexpressing a non-oligomerizable variant of human KCNB1 (Tg-C73A) exhibit reduced tissue damage and improved behavioral outcome following TBI compared to non-Tg mice or transgenic mice expressing the wild type (WT) KCNB1 channel. Moreover, those effects can be neutralized by Dasatinib, a Src inhibitor, which directly impinges the downstream effectors of oxidized KCNB1 channels, the Src tyrosine kinases^7^. Given the significant presence/role of oxidative stress in multiple disease states, it is likely KCNB1 oxidation may be present in conditions beyond TBI.

One such case is Alzheimer’s disease (AD), a dementia characterized by multiple etiologies and pathogenic mechanisms. AD brains exhibit strong evidence of ROS-mediated injury including abnormal levels of protein oxidation, DNA oxidation and lipid peroxidation^8^. Indeed, the oxidative stress hypothesis in Alzheimer’s disease posits that ROS contribute to neurodegeneration and death through the cumulative action of multiple damaging processes. It is not coincidental that KCNB1 channels undergo oxidation in the 3xTg-AD triple transgenic mouse model of AD, where their amounts increase with age^2,9^. Furthermore, non-conducting KCNB1 oligomers cause enhanced calcium spike frequency and decreased Fluo-4 intensity in primary 3xTg-AD neurons^10^. This body of evidence underscores the potentially crucial role of oxidation of KCNB1 channels for AD but also the need to elucidate the impact of this mechanism on AD pathology.

Here, we investigate oxidation of KCNB1 channels in the post mortem human hippocampus and in mouse model of AD. The results of our studies indicate that KCNB1 channels undergo extensive oxidation in the human AD brain along with enhanced phosphorylation of FAK and Src kinases. In the 3xTg-AD brain, KCNB1 oxidation is associated with inflammation and oxidative stress which act in concert to increase intraneuronal β-amyloid. These cellular injures correlate with behavioral deficit,suggesting that oxidation of KCNB1 channels may contribute to human AD pathology.

## Results

### KCNB1 undergoes oxidation in the human brain

**Fig. 1:**
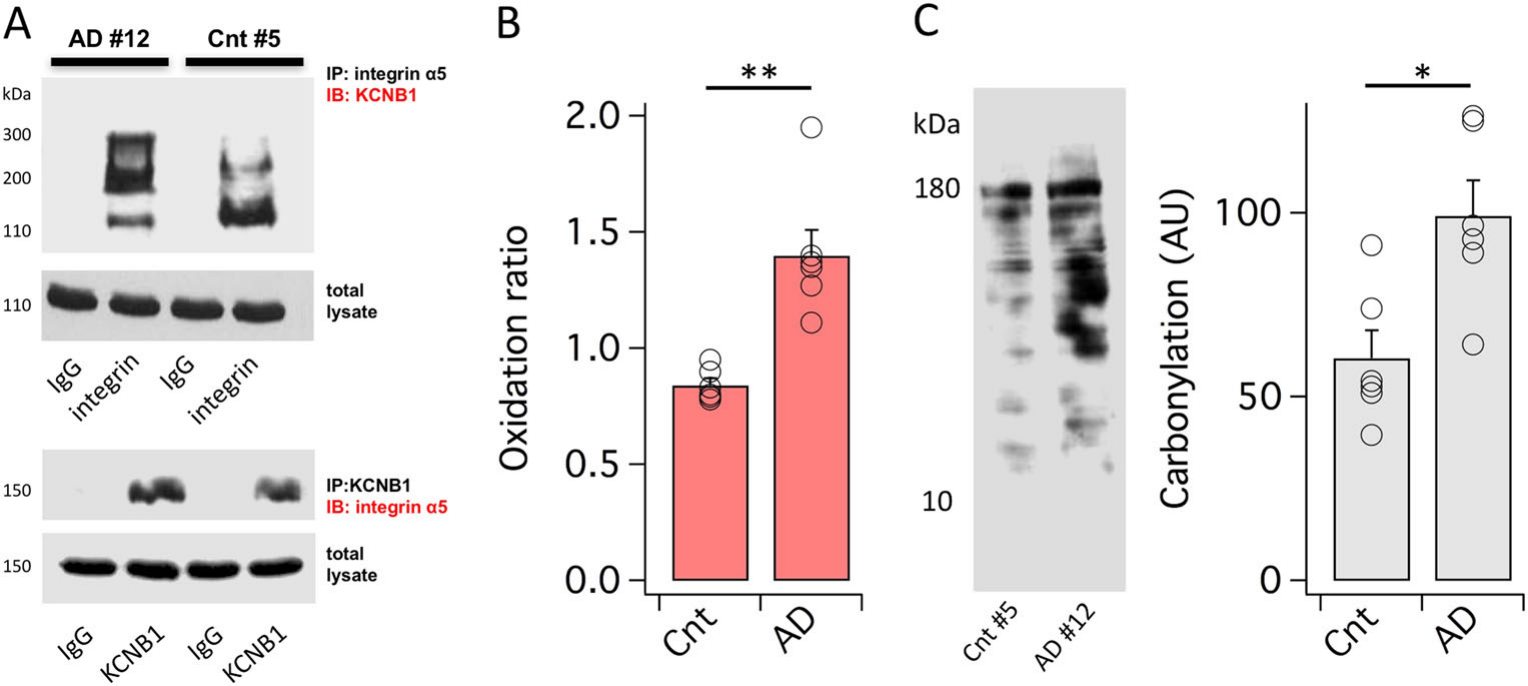
KCNB1 undergoes oxidation in the human brain. **a** In the upper western blots, integrin-α_5_ immunoprecipitates (IP) and IgG IPs (control) from AD sample #12 and control sample #5 and total lysates were immunoblotted (IB) with Kv2.1 primary antibody. Total lysates were treated with 2% β-mercaptoethanol added in the sample buffer and thus do not show oligomers. Integrins pull down both single KCNB1 subunits (∼110 kDa) and oligomers (∼200 to ∼400 kDa). In the lower western blots, KCNB1 and IgG IPs and total lysates were stained with integrin-α_5_ primary antibodies. **b** Mean oxidation ratio from six control samples and six AD samples. Each point represents the average of two technical replicates. **P = 0.008. **c** Representative western blot of protein carbonylation (polyclonal dinitrophenyl (DNP) antibody) and mean protein carbonylation (arbitrary units) from six control and six AD samples. Each point represents the average of two technical replicates. *P = 0.011

KCNB1 forms macromolecular complexes with integrins in the mouse brain^4^. These interactions are retained during the formation of KCNB1 oligomers. In fact, it is the oligomerization of KCNB1 channels that triggers integrin signaling leading to the recruitment/activation of Src tyrosine kinases via autophosphorylated FAK at Tyr397^4^. To assess the mechanism of KCNB1 oxidation in the human brain we obtained post mortem hippocampal tissue of 6 AD donors (3 females and 3 males, average age 83.8 years) and 6 age-matched controls (3 females and 3 males, average age 82.5 years) from the Harvard Brain Tissue Resource Center through the Neurobiobank repository of the NIH. Donors’ information including neuropathology reports is listed in Table 1. To determine whether KCNB1 and integrins form complexes in the human brain, proteins were immunoprecipitated (IP) with an antibody that detects integrin alpha chain V (integrin-α5) and immunoblotted (IB) with an antibody that detects KCNB1 or vice versa. Representative western blots (WB) of co-immunoprecipitation experiments (co-IP, *N* = 3) in the post mortem hippocampus of AD donors and age-matched controls are shown in Fig. 1a. Integrin-α5 pulled down KCNB1 channels and similarly, KCNB1 channels pulled down integrin-α5 suggesting that KCNB1 and integrins form stable complexes in the human hippocampus. A single KCNB1 subunit runs with a molecular mass ∼110 kDa. KCNB1 oligomers typically exhibit molecular masses ∼200 kDa but they can range up to ∼400 kDa^7^. Notably, KCNB1 oligomers were present in both normally aging and AD brains indicating that KCNB1 undergoes oxidation in the human brain. To quantify the extent of KCNB1 oxidation we calculated the densitometric ratio between the monomeric and oligomeric bands (oxidation ratio) that we measured from western blots of brain lysates stained with KCNB1 antibody (the oxidation ratio only depends on the relative amounts of oligomerized and non-oligomerized protein in the blot). This analysis underscored a significant 67% increase in the oxidation ratio of the AD brain vs. control (Fig. 1b). Furthermore, this increase correlated well with the increase in oxidative stress that was assessed by measuring protein carbonylation (64% increase in AD vs. control, Fig. 1c). Thus, KCNB1 oxidation is a physiological process which occurs in the normally aging human brain and is exacerbated in AD, consistent with the elevated oxidative stress associated with this condition.

**Table 1:** Neuropathology reports of the AD and age-matched control donors that provided the post mortem hippocampal tissue used in this study

Post mortem human samples				
Donor #	Diagnosis	Age	Sex	Neuropathology report
1	Control	83	F	1. Neurofibrillary degeneration, Braak and Braak early stage II. 2. Small defect in optic tract with loss of myelinated fibers. 3. Arteriolosclerosis and atherosclerosis. 4. Mild, autolysis.
2	Control	84	M	1. Neurofibrillary degeneration, Braak and Braak early stage I, with rare non-neuritic neocortical amyloid plaques and rare amyloid angiopathy. 2. Arteriosclerosis and atherosclerosis. 3. Mild Purkinje cells loss. 4. Mild autolysis.
3	Control	85	M	1. Neurofibrillary degeneration, late Braak stage II, with non-neuritic neocortical amyloid plaques. 2. Arteriosclerosis, atherosclerosis, and a remote microhemorrhage in the *substantia nigra*.
4	Control	81	M	1. Neurofibrillary degeneration, Braak stage II, with non-neuritic neocortical amyloid plaques. 2. Arteriosclerosis and atherosclerosis.
5	Control	82	F	1. Neurofibrillary degeneration, Braak stage II, with sparse non-neuritic neocortical amyloid plaques. 2. Small vessel cerebrovascular disease with arteriosclerosis, arteriolosclerosis, and a microinfarct in the *nucleus accumbens*.
6	Control	80	F	1. Sparse non-neuritic neocortical amyloid plaques. 2. Arteriosclerosis and atherosclerosis.
7	Alzheimer’s disease	83	F	1. Alzheimer’s disease, Braak stage VI, with severe amyloid angiopathy. 2. Early (preclinical) Parkinson’s disease (incidental Lewy body disease). 3. Arteriosclerosis and atherosclerosis.
8	Alzheimer’s disease	84	M	1. Advanced Alzheimer’s disease, Braak and Braak stage VI, with severe amyloid angiopathy. 2. Small vessel cerebrovascular disease with Arteriosclerosis, arteriolosclerosis and atherosclerosis, arterial mural necrosis, three microinfarcts in the neocortex, lacunes in the *caudate nucleus* and thalamus, and focal zones of acute ischemic change in neocortex. 3. Moderate autolysis.
9	Alzheimer’s disease	82	F	1. Alzheimer’s disease, Braak stage VI, with amyloid angiopathy. 2. Arteriosclerosis.
10	Alzheimer’s disease	82	F	1. Alzheimer’s disease, Braak stage VI, with amyloid angiopathy and hippocampal sclerosis. 2. Small vessel cerebrovascular disease with severe arteriosclerosis, arteriolosclerosis and atherosclerosis and a microinfarct in the interior temporal cortex.
11	Alzheimer’s disease	85	M	1. Alzheimer’s disease, Braak stage VI, with amyloid angiopathy and mesial temporal sclerosis (hippocampal sclerosis). 2. Arteriosclerosis and atherosclerosis. 3. Early Parkinson’s disease.
12	Alzheimer’s disease	87	M	1. Alzheimer’s disease, Braak stage VI, with amyloid angiopathy. 2. Arteriosclerosis.

Donor number (controls 1–6; AD: 7–12), age in years, diagnosis, and sex are indicated

### FAK and Src phosphorylation are increased in the AD brain

**Fig. 2:**
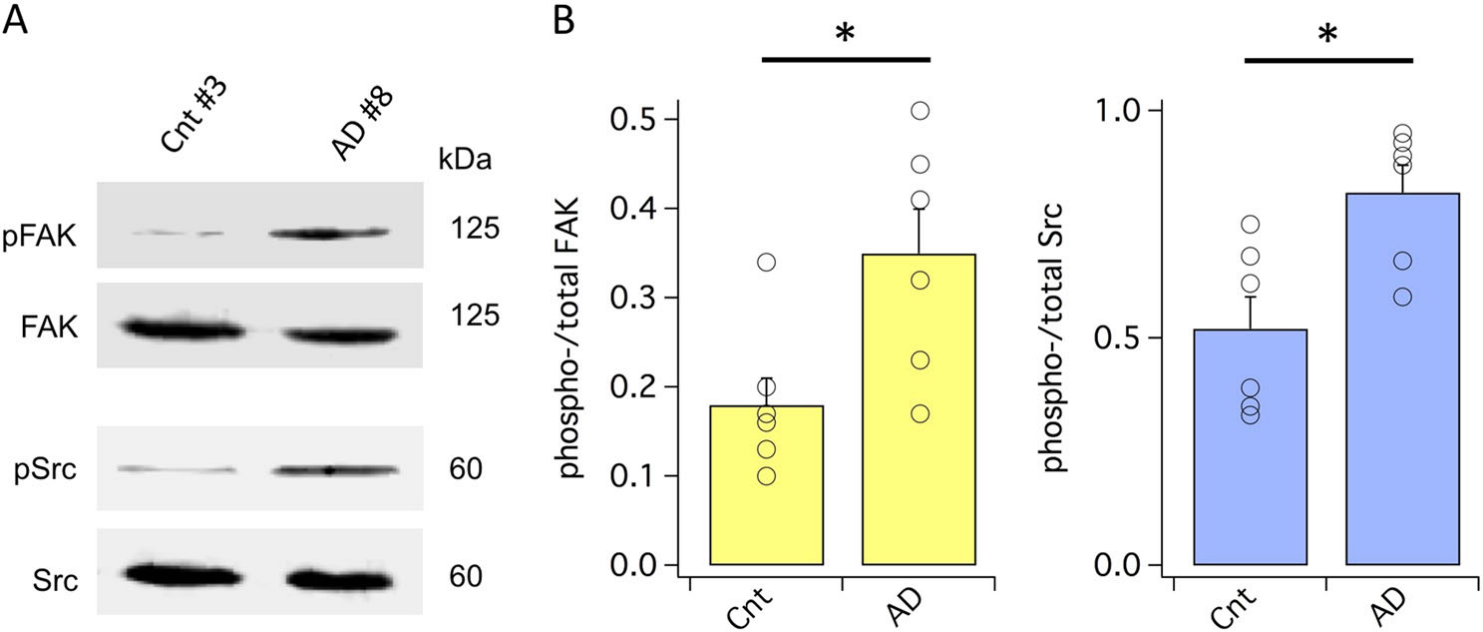
FAK and Src kinases activities are increased in the human AD brain. **a** Representative western blots showing autophosphorylated FAK at tyr397 (pFAK) and total FAK (upper blot) and phosphorylated Src at Tyr 416 (pSrc) and total Src (lower blot) in the hippocampi of AD donor #8 and control donor #3. FAK protein was detected into a single, ∼125 kDa band and Src protein into a single, ∼60 kDa band. **b** Mean fraction of activated FAK (pFAK/FAK) and activated Src (pSrc/tSrc) from six control and six AD samples. Each point represents the average of two technical replicates. *P = 0.028 and *P = 0.013 for FAK and Src, respectively

The representative western blots in Fig. 2a show total and phosphorylated FAK at Tyr397 and Src at Tyr416 in the post mortem brains of an AD donor and of an age-matched control. The fraction of activated FAK and Src was increased by, respectively, 80% and 58% in AD vs. control brains (Fig. 2b), in good agreement with the 67% increase in KCNB1 oxidation/oligomerization.

### KCNB1 is oxidized in mouse model of AD

**Fig. 3:**
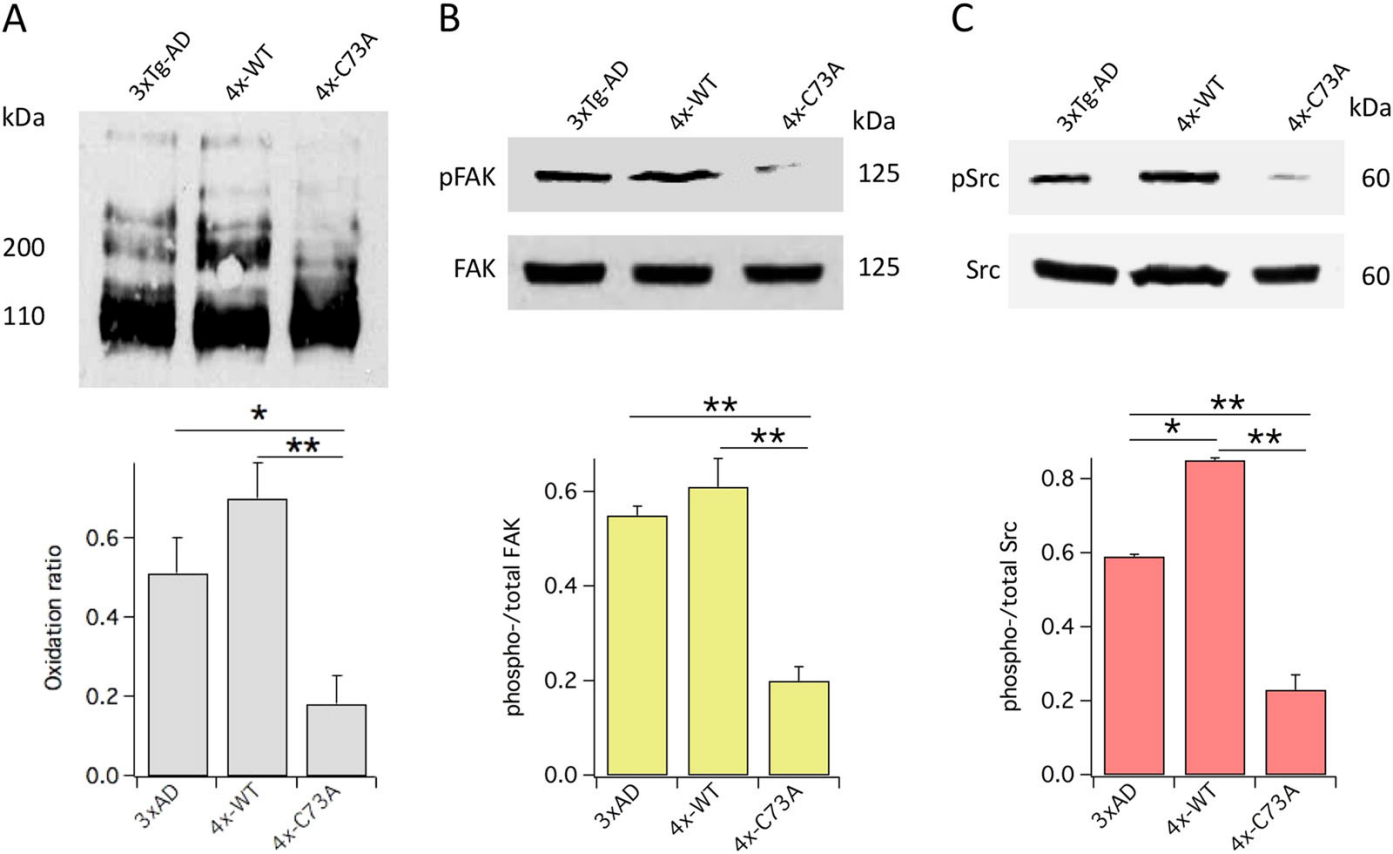
KCNB1 oxidation is recapitulated in mouse model of AD. **a** Representative western blot showing oxidized KCNB1 channels in the brains of 6-month-old 3xTg-AD, 4xTg-WT, and 4xTg-C73A mice and mean oxidation ratio. *N* = 4 brains/genotype. **b** Representative western blots showing autophosphorylated FAK at Tyr397 and total FAK in the brains of 6-month-old 3xTg-AD, 4xTg-WT, and 4xTg-C73A mice and mean pFAK/FAK ratio. *N* = 3 brains/genotype. **c** Representative western blots showing phosphorylated Src at Tyr416 and total Src in the brains of 6-month-old 3xTg-AD, 4xTg-WT, and 4xTg-C73A mice and mean pSrc/Src ratio. *N* = 3 brains/genotype. *P < 0.05 and **P < 0.01

To further elucidate the role of KCNB1 oxidation in AD we studied it in the genetic background of the 3xTg-AD mouse model of AD. This transgenic animal expresses three dementia-related transgenes, namely APP_SWE_, PS1_M146V_, and tau_P301L_ and exhibits both plaque and tangle pathology, as well as synaptic dysfunction^9^. We constructed 4xTg mice by cross-breeding 3xTg-AD with transgenic mice that expresses a non-oligomerizabe variant of KCNB1 obtained by replacing Cys73 to Ala (Tg-C73A) or the WT channel (Tg-WT) as control, in cortex and hippocampus which we characterized previously^7^. We used 4xTg mice heterozygous in either WT or C73A since homozygous Tg-WT mice exhibit developmental delays^7^. Figure 3a summarizes biochemical assessment of KCNB1 oxidation in mouse model of AD. Consistent with results with post mortem human brain tissue, KCNB1 oligomerization was robust in the 3xTg-AD brain and was further increased in mice overexpressing the WT channel. In contrast, KCNB1 oligomerization was low in the brains expressing the C73A variant as expected (we showed previously that exogenous and endogenous KCNB1 subunits form heteromeric complexes. The Cys to Ala mutation is dominant negative and therefore the amounts of oxidized KCNB1 channels are negligible in the Tg-C73A brain^7^). Furthermore, the fractions of phosphorylated FAK kinases at Tyr397 (Fig. 3b) and Src kinases at Tyr416 (Fig. 3c) were significantly increased in 4xTg-WT and 3xTg-AD compared to 4xTg-C73A brains.

### KCNB1 oxidation contributes to cognitive impairment in mouse model of AD

**Fig. 4:**
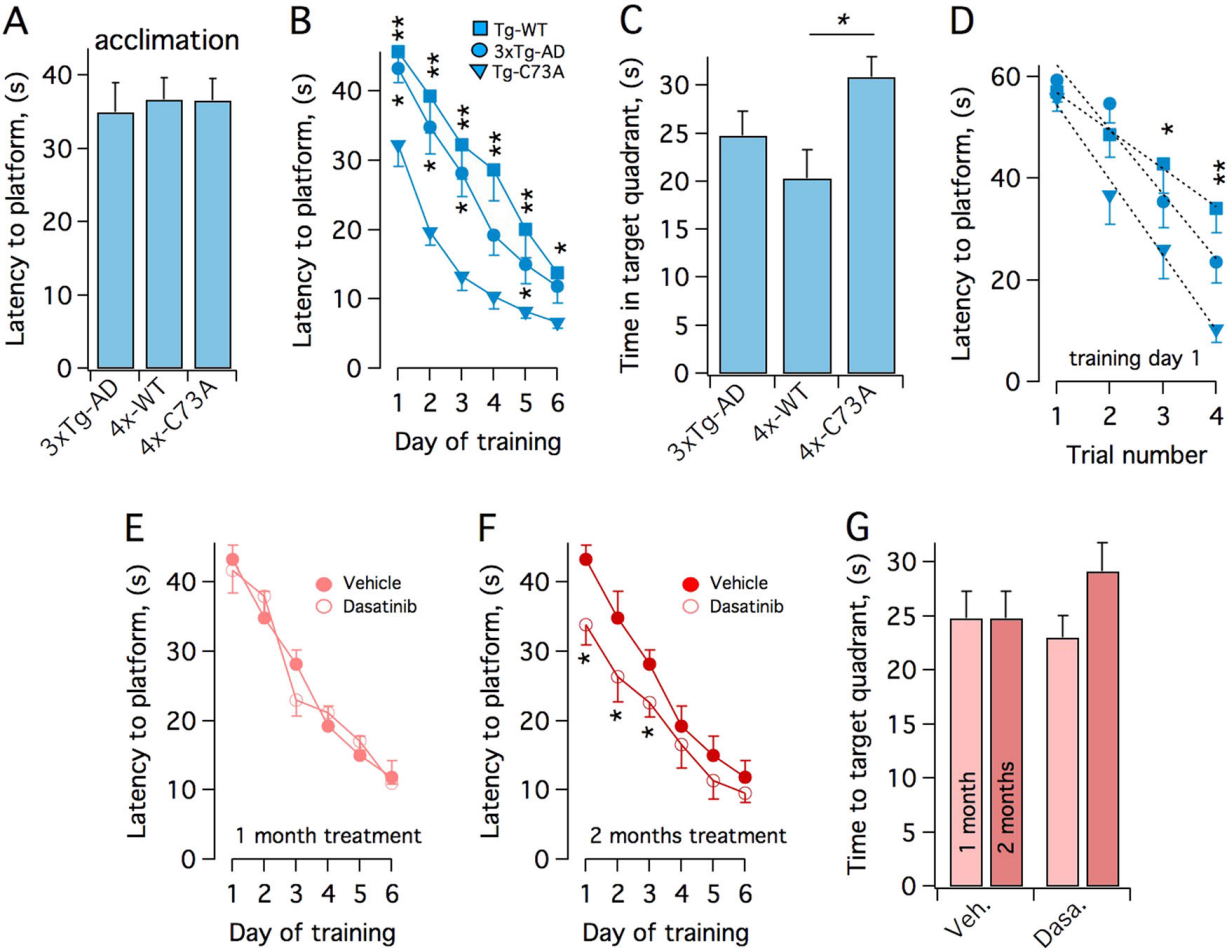
Oxidation of KCNB1 causes working memory impairment in mouse model of AD **a** Mean latency to platform (visible) of the indicated genotypes 4 days before training. **b** Mean latency to platform of 3xTg-AD (circles), 4xTg-WT (squares), and 4xTg-C73A (triangles) mice. **c** Consolidated memory retention test (platform removed) for the indicated groups of mice. **d** Latency to platform of 3xTg-AD (circles), 4xTg-WT (squares), and 4xTg-C73A (triangles) mice, in the individual trials during the first day of training. Fit of the data to a linear function (dotted lines) gave a rate of: −14.72 ± 1.09 s/trial for 4xTg-C73A; −11.67 ± 1.94 s/trial for 3xTg-AD and −7.43 ± 0.36 s/trial for 4xTg-WT mice. **e** Mean latency to platform of 1-year-old 3xTg-AD mice daily ip injected with 25 mg/kg Dasatinib (filled circles) or vehicle (hollow circles) for 1 month. Mean swimming speeds were: 0.37 ± 0.03 and 0.32 ± 0.02 m/s for vehicle and Dasatinib, respectively. **f** Mean latency to platform of 1-year-old 3xTg-AD mice daily ip injected with 25 mg/kg Dasatinib (filled circles) or vehicle (hollow circles) for 2 months. Mean swimming speeds were: 0.35 ± 0.02 and 0.35 ± 0.02 m/s for vehicle and Dasatinib, respectively. **g** Consolidated memory retention test for the indicated groups of mice. In **a** through **d** *N* = 11 mice/genotype. In **b** and **c**, statistical significance of pairwise comparisons between 4xTg-C73A and the other genotypes is indicated. In **e** through (**f**) *N* = 9 mice/genotype. *P < 0.05 and **P < 0.01

To determine the impact of KCNB1 oxidation on the cognitive function of the hippocampus, we assessed 1-year-old mice in the spatial working memory task in the Morris Water Maze (MWM), which is one of the most well-modeled aspects of the working memory deficits of AD^11^. All mice behaved similarly during the acclimation trials when the platform was visible (Fig. 4a), and therefore baseline adjustments were not performed. In contrast, during the training period (Fig. 4b) and in the post-training assessment of memory consolidation with the platform removed (Fig. 4c), 4xTg-C73A mice performed significantly better than 3xTg-AD and 4xTg-WT. The superior learning abilities of 4xTg-C73A mice were already apparent at the beginning of the training. During the first day, these mice learned to locate the platform twice as much as fast than 4xTg-WT mice (gaining ∼14 s per trial vs. ∼7 s per trial of the 4xTg-WT mice) even though all mice performed similarly in the first trial, irrespective of the genotype (Fig. 4d). Swimming speeds were comparable in the various groups of mice (0.32 ±  0.02; 0.34 ± 0.02, and 0.35 ± 0.02 m/s for 3xTg-AD, 4xTg-WT, and 4xTg-C73A, respectively), suggesting that the improved outcome in the 4xTg-C73A mice was not due to increased activity and we also did not observe sex-specific differences in the various genotypes^12^. In previous studies we demonstrated that the only distinction between the WT channel and the C73A variant is that the latter does not undergo oxidation in the brain. This leads us to conclude that differences between the 4xTg and 3xTg genotypes mainly depended on the oxidation of the channel and not on other factors^4,7^ and thus that there is a causal relationship between the extent of KCNB1 oxidation and spatial working memory in mouse model of AD.

### Dasatinib ameliorates AD-like pathology in 3xTg-AD mice

The activation of Src tyrosine kinases is a key signaling event following the formation of KCNB1 oligomers. In previous studies we identified Dasatinib, a FDA-approved specific inhibitor of Src kinases, blood–brain barrier permeable and pharmacologically active in the brain, as a means to neutralize the toxic effects of KCNB1 oxidation in mouse model of TBI (the drug is also associated with decreased KCNB1 oligomerization)^7,13–23^. Since in both TBI and AD oxidation of KCNB1 channels leads to Src activation Dasatinib should also be effective in the latter. Two groups of 10- and 11-month-old 3xTg-AD mice were daily injected with 25 mg/kg Dasatinib, delivered intraperitoneally (ip), for 2 and 1 months respectively and then tested in the MWM. The mice treated for 1 month did not exhibit appreciable improvement compared to vehicle mice (Fig. 4e). Mice that underwent the 2 months treatment performed moderately better than vehicle mice during the 6-day training period (Fig. 4f) and in the memory consolidation test with the platform removed (Fig. 4g). Overall, the drug was less effective in 3xTg-AD mice than in TBI mice, where it could largely suppress the effects of the injury^7^ probably because at the time of the treatment, the AD-like pathology was already at an advanced stage.

### KCNB1 oxidation is associated to astrocytosis in the 3xTg-AD brain

**Fig. 5:**
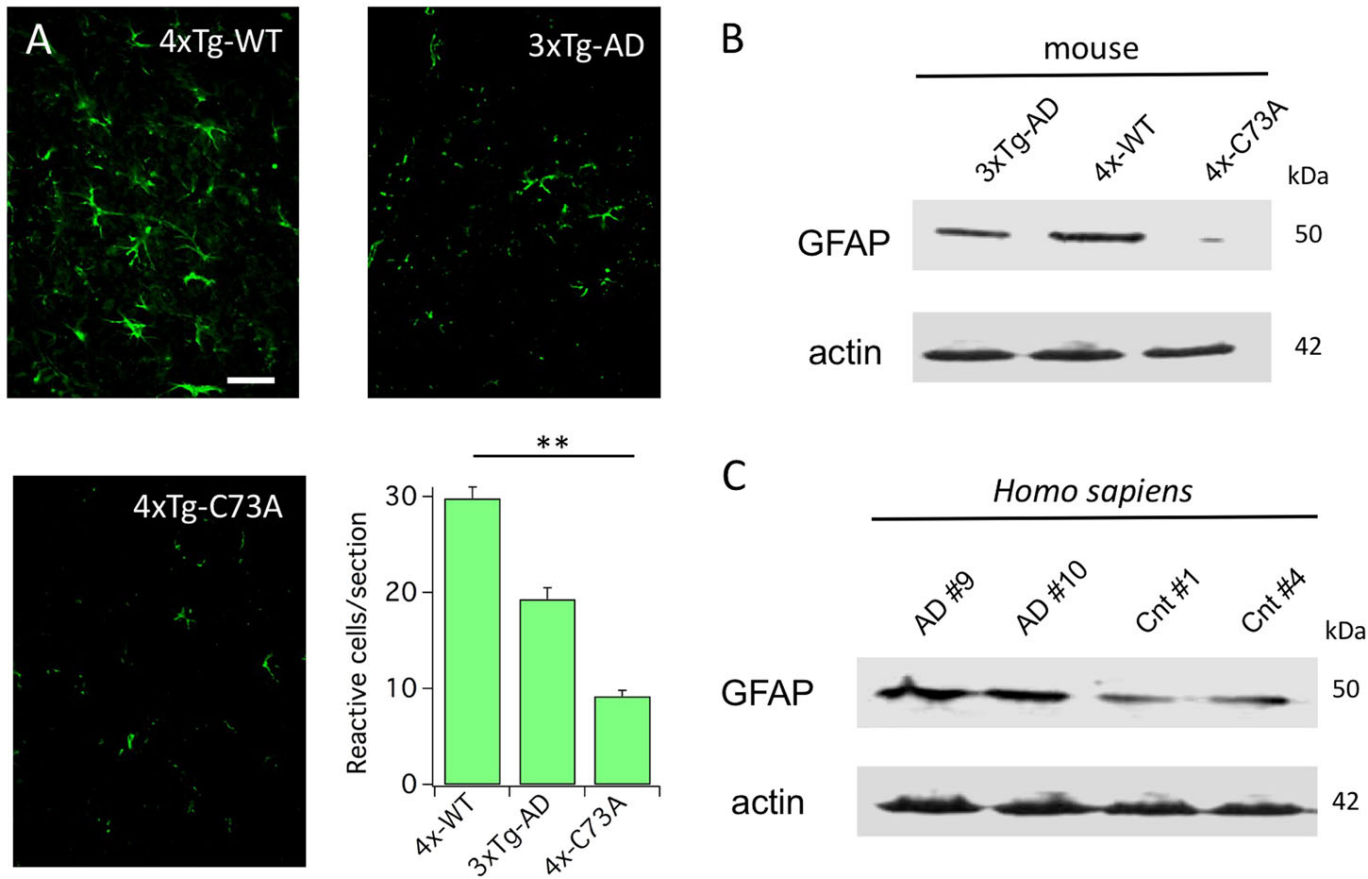
KCNB1 oxidation increases astrocytosis in mouse model of AD. **a** Representative images of coronal hippocampal sections from the brains of the indicated genotypes stained with GFAP antibody and mean number of cells reactive for GFAP, per section. Scale bar: 200 μm. Each single mean was calculated from 12 sections (3 brains, 2 fields of view/section). **b** Representative western blot of GFAP protein in the brains of the indicated genotypes. **c** Representative western blot of GFAP protein in the brains of AD donors #9 and #10 and age-matched controls #1 and #4. **P *<* 0.01

Astrocytosis is broad in AD, and glial fibrillary acidic protein (GFAP) is a useful biomarker for the disease^24–26^. We assessed the levels of GFAP in the brain by immunohistochemistry (IHC, Fig. 5a) and western blot (Fig. 5b). Reactive astrocytes were more numerous in the hippocampi of 4xTg-WT and 3xTg-AD compared to 4xTg-C73A, a result corroborated by western blot analysis. Similarly, the amounts of GFAP protein were increased in post mortem AD brains compared to age-matched controls, as expected (Fig. 5c)^24,25^.

### Oxidation of KCNB1 contributes to inflammation and oxidative stress in the 3xTg-AD brain

**Fig. 6:**
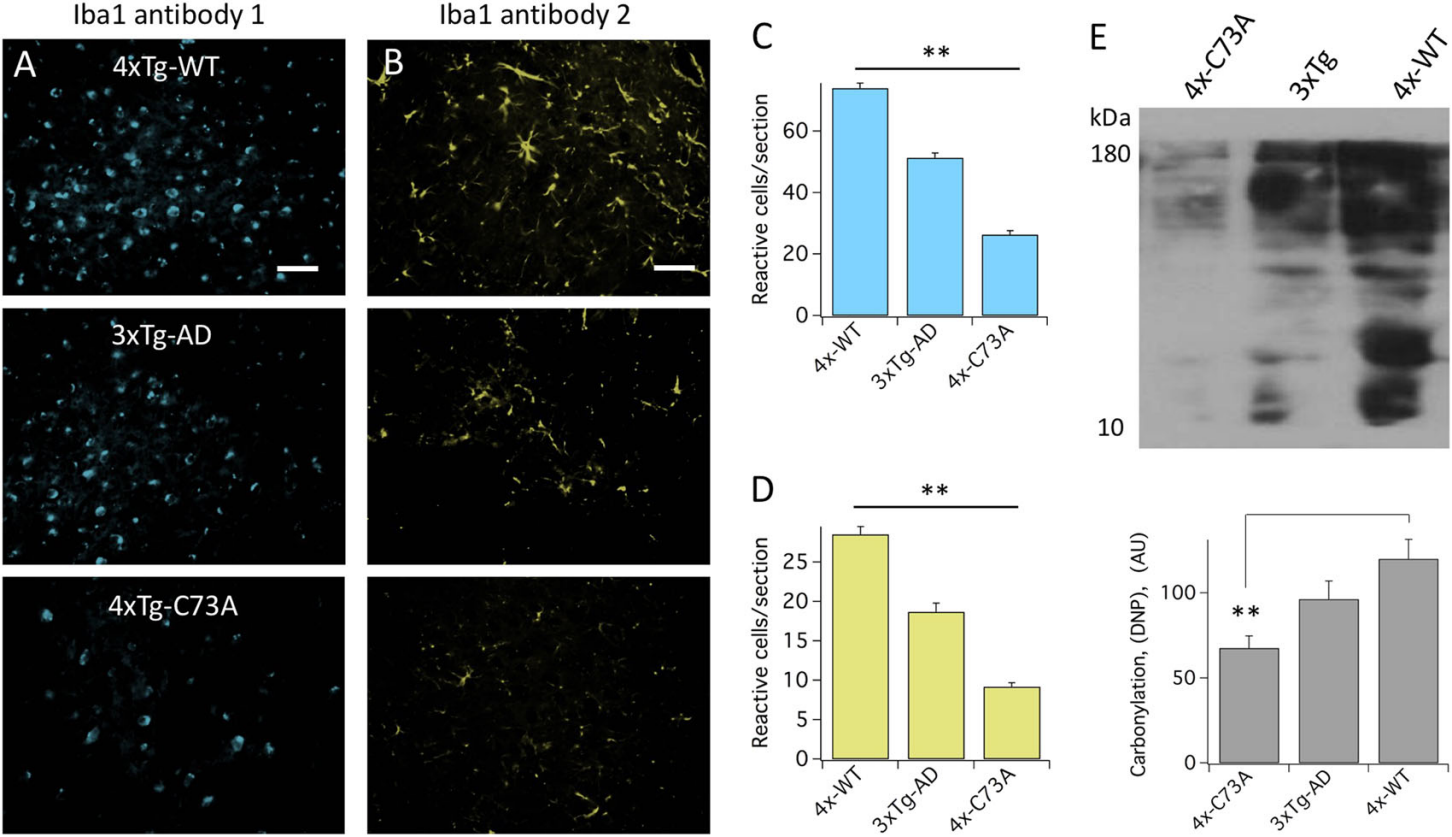
KCNB1 oxidation increases inflammation in mouse model of AD. **a**, **b** Representative images of coronal hippocampal sections from the brains of the indicated genotypes stained with an antibody against Iba1 from Abcam (**a**) or from Wako (**b**). Scale bar 100 μm. **c**, **d** Mean number of cells reactive to Iba1 Abcam (**c**) or Wako (**d**) antibody per section for the indicated genotypes. Each mean was calculated from 12 sections (3 brains, 2 fields of view/section). **e** Representative western blot of protein carbonylation and mean protein carbonylation (arbitrary units) in the brains of the indicated genotypes. *N* = 3 brains/genotype. **P < 0.01

Astrocytosis generally accompanies inflammation, thus GFAP provides indirect evidence for inflammation^27,28^. We directly assessed inflammation by staining hippocampal sections with two antibodies (Abcam clone ab5076, Fig. 6a, c and Wako clone 019–19741, Fig. 6b, d) against allograft inflammatory factor 1 (Iba1). Reactive microglia reactive for Iba1 were present in the hippocampus of 3xTg-AD mice (Fig. 6a, b). Reactive cells were increased in 4xTg-WT sections and were significantly decreased in 4xTg-C73A sections (Fig. 6c, d). The antibodies stained accordingly to manufacturer’s datasheets (clone ab5076: globular cells; clone 019–19741: branched cells); however, due to the shared lineage of microglia and macrophages, markers are common to both cell types. Therefore we cannot rule out that reactive cells are composed of both cell types, but we can reasonably conclude that inflammation is decreased in the 4xTg-C73A brain compared to the other genotypes.

### Oxidative stress is low in neurons expressing C73A

The pathway activated in response to KCNB1 oligomerization promotes oxidative stress, a hallmark of AD^3,7^. Accordingly, protein carbonylation, which provides a measure of oxidative stress, was markedly lessened in the brains of 6-month-old 4xTg-C73A mice compared to 3xTg-AD and 4xTg-WT, where it was maximal (Fig. 6e).

### Oxidation of KCNB1 contributes to amyloidosis

**Fig. 7:**
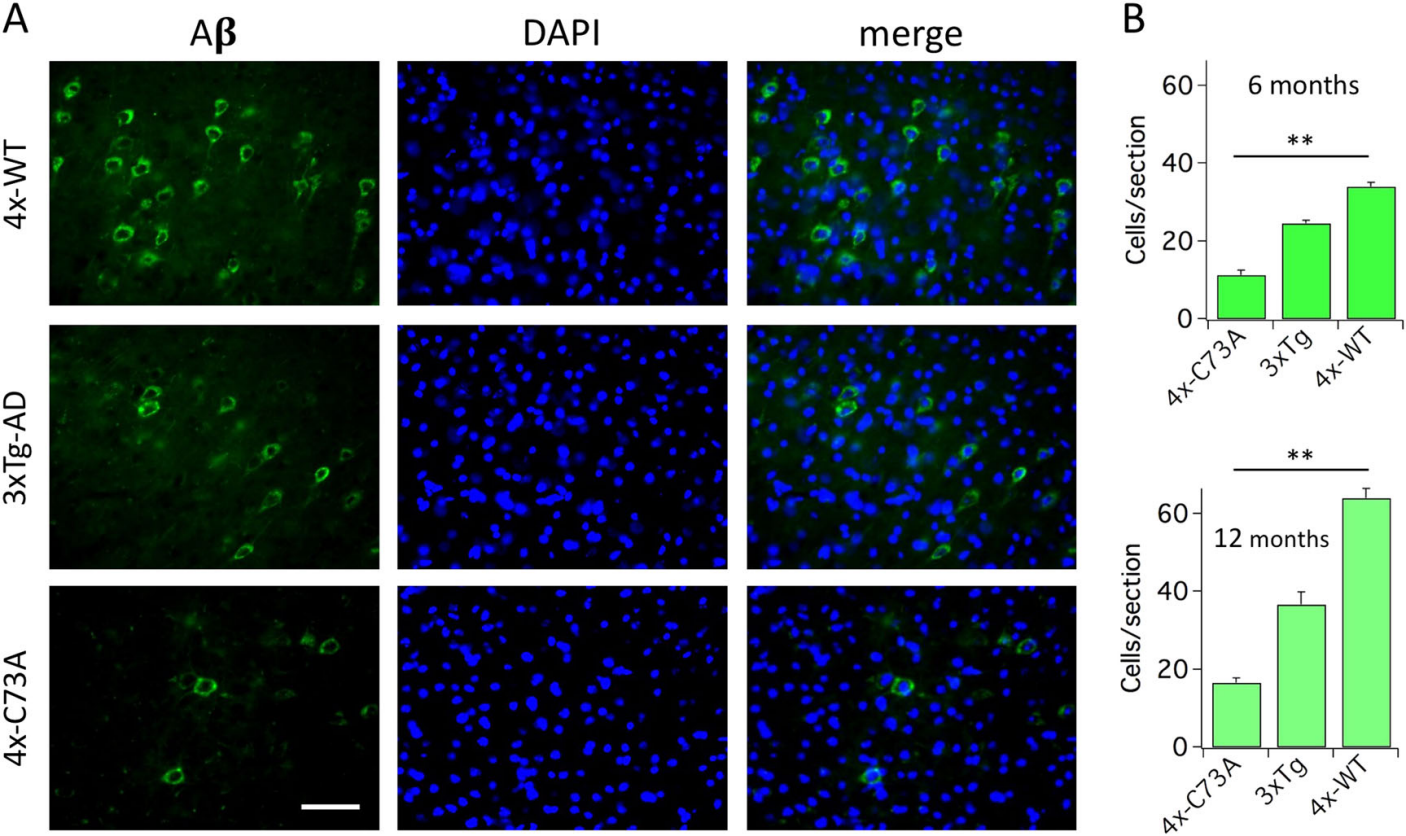
KCNB1 oxidation is associated with increased intraneuronal Aβ in young mice. **a** Representative images of coronal hippocampal sections from the brains of the indicated 6-month-old genotypes stained with human Aβ antibody and DAPI. Scale bar: 40 μm. **b** Mean number of neurons reactive for Aβ per section in the hippocampi of 6-month-old (green) and 12-month-old (pale green) mice of the indicated genotypes. Each mean was calculated from 12 sections (3 brains, 2 fields of view/section)

**Fig. 8:**
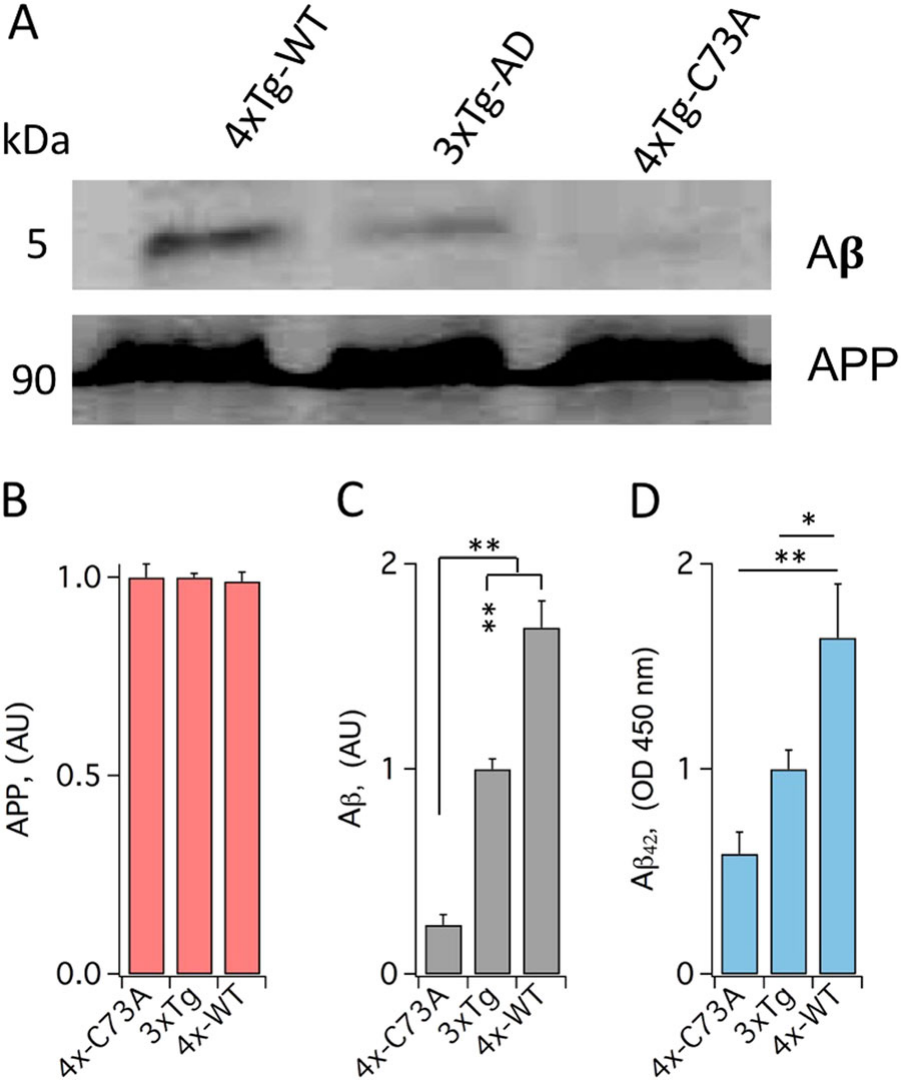
KCNB1 oxidation contributes to increase Aβ oligomers. **a** Representative western blots showing Aβ protein and APP protein in the brains of the indicated 1-year-old genotypes. **b** Densitometric quantification of APP protein by western blot analysis (as shown in **a**) in the brains of the indicated 1-year-old genotypes. Data are normalized to 3xTg-AD. *N* = 3 brains/genotype. **c** Densitometric quantification of Aβ protein by western blot analysis (as shown in **a**) in the brains of the indicated 1-year-old genotypes. Data are normalized to 3xTg-AD. *N* = 3 brains/genotype. **d** ELISA quantification of Aβ(1–42) in the brains of the indicated 1-year-old genotypes. Data are normalized to 3xTg-AD (1 = 3.2 pg/ml). *N* = 6 brains/genotype. *P < 0.05 and **P < 0.01

Neuroinflammation and oxidative stress sustain the synthesis of β-amyloid (Aβ)^29^. The 3xTg-AD mice develops intracellular Aβ in the hippocampus at around 6 months of age and plaque around 1 year^9^. Indeed, neurons reactive for intracellular Aβ were more numerous in hippocampal sections cut from the brains of 6- and 12-month-old 4xTg-WT and 3xTg-AD mice compared to 4xTg-C73A mice (Fig. 7a, b). We assessed amyloid precursor protein (APP), Aβ and Aβ(1–42) protein by Western blot and enzyme-linked immunosorbent assay (ELISA) analysis, respectively, in the brains of 1-year-old mice. While the amounts of APP were similar in all genotypes (Fig. 8a, b) the amounts of Aβ protein (Fig. 8a, c), including the most neurotoxic Aβ(1–42) form (Fig. 8d), were significantly lower in the C73A brains compared to the 3xTg-AD and 4xTg-WT brains. Aβ(1–42) was also moderately decreased (29% reduction) in the brains of 3xTg-AD mice treated with Dasatinib for 2 months (3.4±0.4 and 2.4±0.1 pg/ml in the absence/presence of Dasatinib, *P* < 0.043 one-tailed Student’s *t*-test, *N* = 3 brains/group, data not shown). In addition, the number of neurons reactive for Aβ was increased by 88% in 4xTg-WT brains between 6 and 12 months of age but only by 46% in 4xTg-C73A brains (Fig. 7b) suggesting that oxidation of KCNB1 exhibits an accelerating influence on the production of Aβ. Taken together, these data underscore a mechanistic link between oxidation of KCNB1 and the production of Aβ and further suggest that KCNB1 oligomers impact the pathogenesis of Aβ early.

### KCNB1 oxidation contributes to tauopathy

**Fig. 9:**
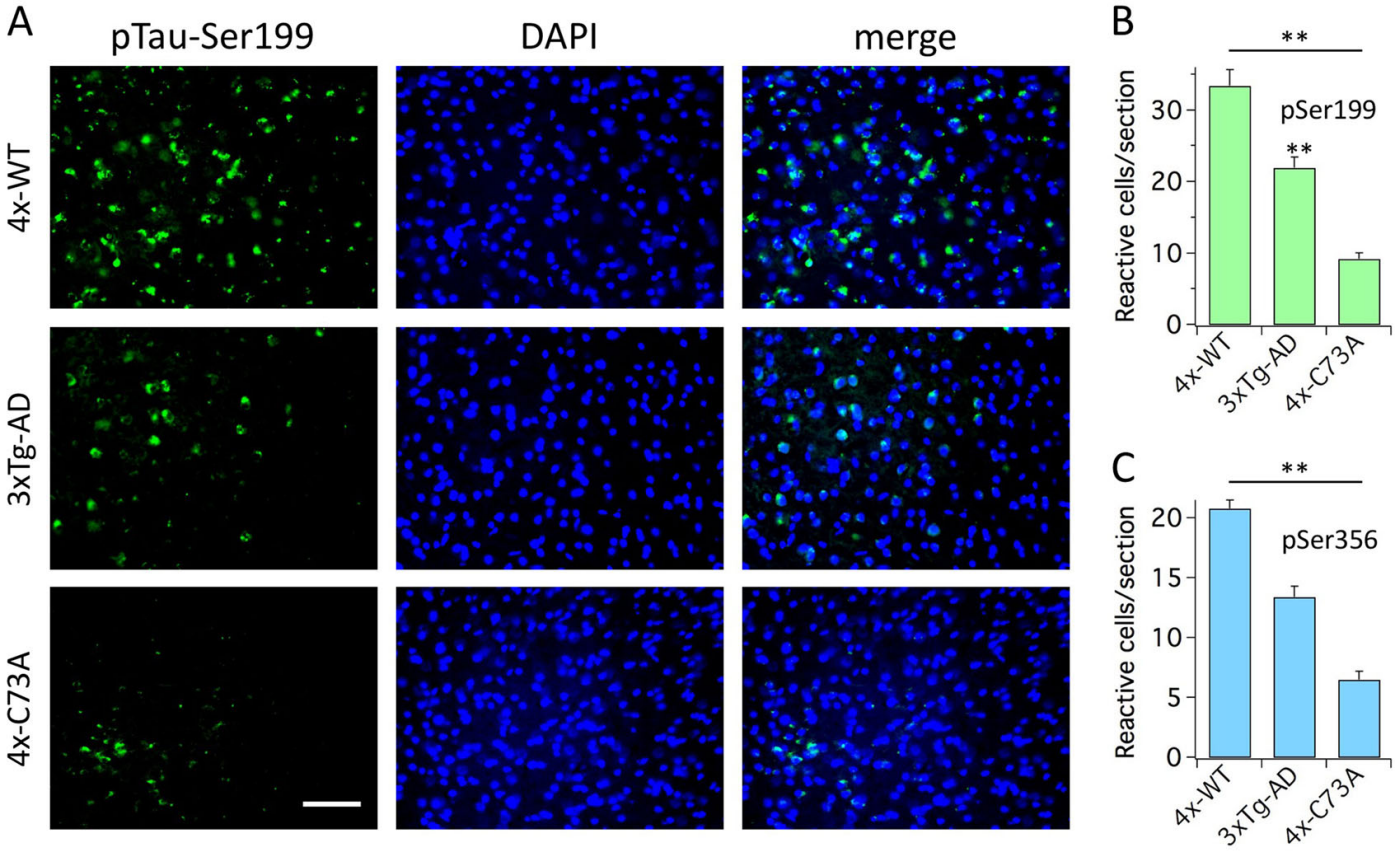
KCNB1 oxidation contributes to tauopathy. **a** Representative images of coronal hippocampal sections from the brains of the indicated 1-year-old genotypes stained with an antibody that detects tau protein phosphorylated as Ser199 and DAPI. Scale bar: 40 μm. **b** Mean number of neurons reactive for an antibody that detects tau protein phosphorylated at Ser199 per section in the hippocampi of 1-year-old mice of the indicated genotypes. Each mean was calculated from 12 sections (3 brains, 2 fields of view/section). **c** Mean number of neurons reactive for an antibody that detects tau protein phosphorylated at Ser356 per section in the hippocampi of 1-year-old mice of the indicated genotypes. Each mean was calculated from 12 sections (3 brains, 2 fields of view/section). **P < 0.01

It has been proposed that Aβ may contribute to tau hyperphosphorylation and evidence further shows that oxidative stress is interlinked with tauopathy^30–36^. Since oxidation of KCNB1 is associated to both amyloidosis and oxidative stress, we assessed the levels of hyperphosphorylated tau in the brains of 1-year-old mice by IHC, using an antibody that detects phosphorylated tau at Ser199 (Fig. 9a, b), and phosphorylated tau at Ser356 (Fig. 9c). The number of reactive neurons to both antibodies was maximal in 4xTg-WT hippocampi and minimal in 4xTg-C73A hippocampi. Tauopathy was less advanced than amyloidosis or inflammation, consistent with the fact that in the 3xTg-AD mouse it develops around 1 year of age, which was the maximal age reached by our animals^9,33^.

## Discussion

Reactive oxygen species and K^+^ channels hold key roles in both the physiology and pathology of the brain. Evidence of oxidation of K^+^ channels in TBI as well as AD is suggestive of a general mechanism of neuronal vulnerability that can potentially affect all conditions characterized by oxidative stress, from normal aging to neuropathies^37,38^. To address this fundamental question we investigated oxidation of KCNB1 channels in AD, a condition associated to massive oxidative stress. Our studies reveal the presence of oxidation-induced KCNB1 oligomers in post mortem hippocampal tissue of aged human donors and, to a significantly larger extent, of AD donors. Phosphorylation of FAK and Src kinases—two key signaling events triggered by KCNB1 oxidation/oligomerization--are also markedly increased in the AD brains compared to age-matched controls. The possibility exists that KCNB1 channels had undergone oxidation during the preparation of the samples, for example, during the time elapsed between the death of the donor and the collection of the tissue. However, these technical issues should have affected the samples randomly and thus alone, cannot explain the significant differences in the extent of KCNB1 oxidation, protein carbonylation, FAK and Src phosphorylation and GFAP immunoreactivity that exist between control and AD brains. Further, oxidized KCNB1 channels are detected in the brains of normally aging mice and, in significantly larger quantities, in 3xTg-AD brains where these technical issues are minimized^2^. We conclude that oxidation of KCNB1 channels is a process that takes place in the human brain under pathophysiological conditions.

To gain mechanistic insight into the role of KCNB1 oxidation in AD, we engineered 3xTg-AD mice to overexpress either the C73A variant (low oxidation) or the WT channel (high oxidation) in cortex and hippocampus. Increased amounts of KCNB1 oligomers correlated with increased amounts of GFAP and Iba1, two markers for astrocytosis and inflammation, along with FAK and Src phosphorylation and protein carbonylation. At the behavioral level, 4xTg-C73A mice performed significantly better than the other genotypes in the working memory task of the MWM. KCNB1 oxidation promotes inflammation, oxidative stress, neuronal apoptosis, and behavioral deficit in mouse model of traumatic brain injury a condition that, likewise AD, is associated to copious release of ROS^7^. Hence, oxidative stress contributes to neurotoxicity via modification of K^+^ channels irrespective of the specific pathology. We conclude that oxidation of K^+^ channels is a general mechanism of neuronal vulnerability that is conserved from worms to humans^37^.

Previous studies have implicated oxidized KCNB1 channels in AD by showing that they induce hyperexcitability in primary 3xTg-AD hippocampal neurons (oxidized KCNB1 channels do not conduct current)^2,10^. The work presented here adds new insight into the molecular mechanisms underlying KCNB1′s neurotoxicity in AD. Thus, neuroinflammation and oxidative stress are two well established factors that contribute to the production of Aβ. In turn, elevated levels of Aβ can create a positive feedback loop toward generating more oxidative stress. Accordingly, in the 4xTg-WT brain, where KCNB1 oxidation is exacerbated, neuroinflammation and oxidative stress are increased and intraneuronal Aβ is also increased, whereas in the 4xTg-C73A brain, where KCNB1 oxidation is diminished, intraneuronal Aβ is decreased along with neuroinflammation and oxidative stress. A significant reduction of the levels of intraneuronal Aβ can be already observed in 6 month-old 4xTg-C73A mice, an age in which plaque deposition is still absent while oxidative stress and inflammation are already elevated, suggesting that KCNB1 oxidation is an early contributor to Aβ pathogenesis^9,33^. Aβ and oxidative stress may also contribute to induce phosphorylation of tau. Indeed, we observed neurons reactive to hyperphosphorylated tau in all genotypes that following a well established pattern, were more numerous in 4xTg-WT and 3xTg-AD hippocampi compared to 4xTg-C73A. However, the effects of KCNB1 oxidation on tauopathy will need further scrutiny as in the 3xTg-AD mouse this begins around 1 year of age, the maximal age reached by the mice used in this study^9,33^. Overall, these results are in agreement with previous studies. One of them in particular, from Rehamn and colleagues showed that inhibition of JNK kinases, which are one of the end-points of the signaling pathway activated by oxidized KCNB1 channels results in marked reductions of Aβ production and hyperphosphorylated tau along with inflammation, apoptotic neurodegeneration and improved behavioral outcomes^3,39^.

Dasatinib, which shows robust efficacy in mouse model of TBI by directly impinging on the pathway activated by KCNB1 oxidation and in the APP/PS1 mouse model of AD, had only modest effects in preventing the AD-like pathology of the 3xTg-AD mouse^7,18^. It is likely that oxidation of KCNB1 occurs during the early stages of the disease and thus treatments that impinge on this mechanism need to be started early. Accordingly, the drug exhibited increased efficacy in younger animals subjected to longer treatment. Dasatinib is blood-brain barrier permeable and is the first line drug for patients with Philadelphia chromosome-positive Central Nervous System (CNS) leukemia^13–15,17^ and CNS chronic myeloid leukemia^16,19,20,22,23^. A factor that can limit Dasatinib’s accumulation in the brain is P-glycoprotein (P-gp, ABCB1), as ABCB1 knock out mice exhibit significantly higher amounts of Dasatinib in the brain compared to control^40–43^. However, P-gp function decreases in the aging brain and is severely compromised in the AD brain^44–47^. Thus, Dasatinib therapy may represent a possible strategy for the treatment of AD.

## Conclusions

- This study provides the first experimental evidence that oxidative modification of KCNB1 takes place in the aging human brain and is exacerbated in the Alzheimer’s brain.
- KCNB1 oxidation, cause neuroinflammation, amyloidosis, and cognitive impairment.
- Src kinase inhibitor Dasatinib may represent a possible strategy for the treatment of AD.

## Materials and methods

### Reagents

Src (clone 2108), phospho-Src at tyr416 (clone 2101), FAK (clone 3285), and phospho-FAK at tyr397 (clone 3283) antibodies were purchased from Cell Signaling Technology (Danvers, MA). Kv2.1 antibody (clone K89/34) was purchased from NeuroMab, UC Davis/NIH. GFAP antibody (clone MAB3402) and actin antibody (clone MAB1501) were purchased from Millipore, Billerica, MA. Human β-amyloid antibody (clone 6E10) was purchased from Biolegend, San Diego, CA. Iba1 antibodies were purchased from Abcam (Cambridge, MA, clone ab5076) and from Wako Chemicals (Richmond, VA, clone 019–19741). Phospho-tau antibody (Ser199) clone 2H23L4, was purchased from ThermoFisher Scientific (Waltham, MA) and phospho-tau antibody (Ser356) clone ab75603, was purchased from Abcam. Integrin-α5 antibody (sc-10729, now discontinued) and goat anti-rabbit IgG-R (clone sc-2091) were purchased from Santa Cruz Biotechnology (Dallas, TX). Amyloid-beta 42 ELISA kit was purchased from ThermoFisher Scientific and used following manufacturer’s instructions. Protein Carbonyl Immunoblot kit was purchased from Cell Biolabs, Inc, San Diego, CA and used following manufacturer’s instructions.

### Transgenic mice

We obtained 4xTg-AD transgenic mice heterozygous in KCNB1 by cross-breeding 3xTg-AD mice with Tg-WT (4xTg-WT) or Tg-C73A (4xTg-C73A) mice that we previously characterized^4,7^. Briefly, Tg-WT and Tg-C73A mice overexpress human WT or C73A KCNB1 tagged to the human influenza hemagglutinin (HA) tag in the C-terminus in cortex and hippocampus driven by the *thy1.2* cassette^48^. These mice have been donated to the MMRRC repository of the NIH and are available from them (MMRRC:43829 and MMRRC:43830). In heterozygous mice, the amounts of human KCNB1 protein are comparable in the two transgenic lines and are about half the amounts of endogenous KCNB1 protein. Primary hippocampal neurons express functional KCNB1 currents, whose steady-state amplitudes at +80 mV are comparable in Tg-WT and Tg-C73A transgenic neurons and roughly ~40% larger than in non-Tg neurons. The Cys73 to Ala replacement in the C73A variant does not affect channel’s functional attributes nor its ability to cluster in the plasma membrane.

### Biochemistry

The detailed biochemical procedure was previously described^4,49^.

#### Immunoblots

Approximately 100 mg of human frozen hippocampal tissue (a gift of the Harvard Brain Tissue Resource Center) or half sagittal mouse brains of either sex were homogenized with a glass tissue grinder in lysis buffer [0.32 M sucrose, 5 mM Tris-Cl pH 6.8, 0.5 mM EDTA, 1 mM PMSF, and protease inhibitor cocktail set I (Calbiochem, San Diego, CA)]. Samples were centrifuged at 2000 rpm for 10 min and the supernatant used for biochemical analysis. Protein content was quantified with the Bradford colorimetric assay (Sigma, St. Louis, MO) and dissolved in Laemmli buffer with or without reducing agents. Proteins were resolved by 8–12% SDS-PAGE and transferred to a PVDF membrane that was incubated in a 5% solution of nonfat milk in Tween 20-PBS (PBST) for 2 h at room temperature. After overnight incubation at 4 °C with the primary antibody, the membrane was washed for 20 min and incubated at room temperature with the appropriate secondary antibody.

#### Co-immunoprecipitations

Brain lysates were incubated at 4 °C overnight in the presence of Kv2.1 or integrin-α5 or IgG antibodies. Then, protein A agarose beads (30 μl of 50% bead slurry) were added and incubated overnight at 4 °C. Samples were centrifuged for 30 s at 4 °C and the pellet was washed five times in cell lysis buffer. The pellet was resuspended with 50 μl 2× SDS Laemmli buffer, heated at 100 °C for 10 min and centrifuged for 1 min at 14,000 × *g*. The samples were loaded on 8% SDS-PAGE gel and immunoblotted. The blots were washed in PBST for 20 min and incubated for 5 min with chemiluminescence substrates and exposed.

Densitometry analysis was performed using ImageJ (NIH) software.

### Immunohistochemistry

Mice were transcardially perfused with 0.9% saline followed by 4% paraformaldehyde. The brains were extracted and cryoprotected in 30% sucrose. Twenty micrometers frozen sections were prepared throughout the cortex and the hippocampus in a 1:20 series so that the same set of tissue samples could be used for expression of different makers. Slides were incubated overnight in with antibody at 4 °C (GFAP 1:500, all other antibodies 1;100). Slides were then incubated in the appropriate secondary antibody. All slides were mounted in VECTASHIELD Antifade Mounting Medium (Vector Laboratories, Burlingame, CA) and stored at 4 °C. Staining was visualized on a Zeiss Axiophot microscope at ×40. Positive were counted in coronal sections representing a 1:20 series inclusive of the entire length of the hippocampus. CA1 and CA3 as well as the dentate gyrus was used for quantitation of cells in the hippocampus.

### Morris water maze

The detailed procedure for the MWM was previously described^7^. Briefly, mice were acclimated to the paradigm and tested for baseline response using a visible platform test 4 days prior. The animals were placed in a circular pool of water containing non-toxic white paint and a clear platform for escape. To assess learning, mice were trained using a hidden platform fixed in one of four quadrants for 6 consecutive days (4 trials/day). Black and white distal cues were placed on the walls. The quadrant in which the mouse was placed was pseudo-randomly varied throughout training and the time to locate the platform was recorded. Maximum trial time was 60 s and the mouse remained or was placed on the platform for 15 sec and warmed for 10 min between trials. To assess memory retention, the day after the last training session the animals were be subjected to a 60 s probe trial with the platform removed and the time spent in the target quadrant was measured^50^. Data were recorded using a video-tracking system (EthoVision XT; Noldus Information Technology, Leesburg, VA).

### Drug administration

Dasatinib (LC Laboratories, Woburn, MA) was given intraperitoneally (ip) at 25 mg/kg. Dasatinib was diluted in vehicle solution (50 mM NaAc, pH = 5.0) from a 200 mg/ml stock in dimethyl sulfate (DMSO). Each mouse was subjected to a daily dose of either vehicle or Dasatinib solution via an ip injection.

### Statistical analysis

Quantitative data are presented as mean ± standard error of the mean (SEM). The level of significance, assumed at the 95% confidence limit or greater (*P* < 0.05), was estimated using the Student’s *t-*test (http://studentsttest.com) and one-way ANOVA with a Tukey post hoc test (http://vassarstats.net/anova1u.html).
